# Supplementary figures and images for: Examining the molecular clock hypothesis for the contemporary evolution of the rabies virus
Source: PLoS Pathog. 2024 Nov 25;20(11):e1012740. doi: 10.1371/journal.ppat.1012740 (PMC11627394; doi:10.1371/journal.ppat.1012740)

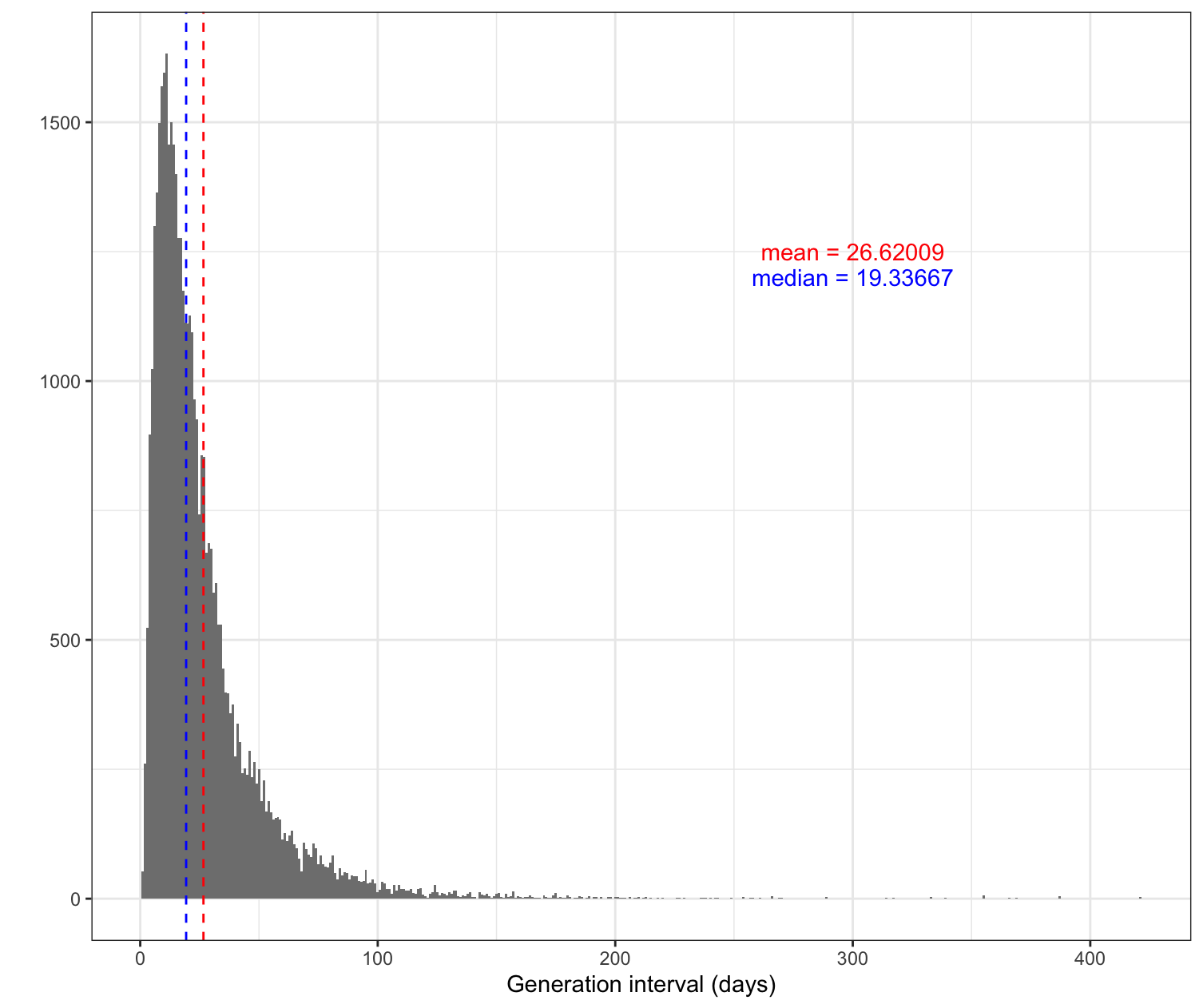

Supplement: S1 Fig — Vertical dashed lines represent the median (blue) and mean (red) generation interval. (TIF) [file ppat.1012740.s001.tif]

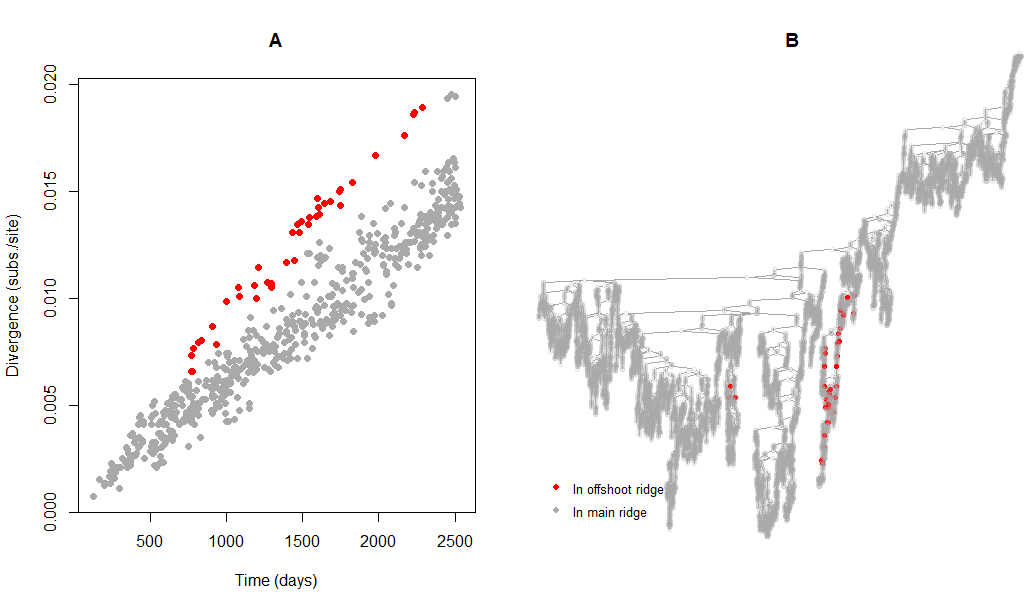

Supplement: S2 Fig — (A) root-to-tip divergence plot (2 SNPs/genome/generation, 5% of cases sequenced) with offshoot ridge points highlighted in red. Offshoot ridge points are defined in this plot as having a divergence rate above 8x10^-6^ substitutions/day and occurring after day 750. (B) transmission tree of the simulated outbreak with offshoot ridge cases highlighted in red. Graph edge length is not proportional to time or divergence. (TIF) [file ppat.1012740.s002.tif]

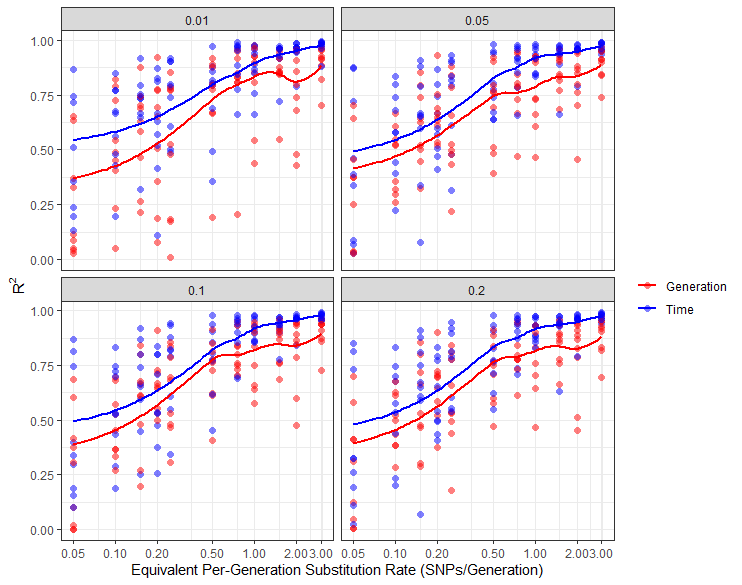

Supplement: S3 Fig — Plot is faceted by the proportion of the total number of cases in the outbreak sequenced, point colour represents mutation model. (TIF) [file ppat.1012740.s003.tif]
